# Sarek: A portable workflow for whole-genome sequencing analysis of germline and somatic variants

**DOI:** 10.12688/f1000research.16665.2

**Published:** 2020-09-04

**Authors:** Maxime Garcia, Szilveszter Juhos, Malin Larsson, Pall I. Olason, Marcel Martin, Jesper Eisfeldt, Sebastian DiLorenzo, Johanna Sandgren, Teresita Díaz De Ståhl, Philip Ewels, Valtteri Wirta, Monica Nistér, Max Käller, Björn Nystedt

**Affiliations:** 1 Department of Oncology-Pathology, Karolinska Institutet, J5:30 BioClinicum, Visionsgatan 4, Karolinska University Hospital at Solna, Solna, 17164, Sweden; 2 Department of Biochemistry and Biophysics, Science for Life Laboratory, Stockholm University, Box 1031, Solna, 17121, Sweden; 3 Department of Cell and Molecular Biology, National Bioinformatics Infrastructure Sweden, Science for Life Laboratory, Uppsala University, Husargatan 3, Uppsala, 752 37, Sweden; 4 Department of Physics, Chemistry and Biology, National Bioinformatics Infrastructure Sweden, Science for Life Laboratory, Linköping University, Linköping, 58183, Sweden; 5 Department of Biochemistry and Biophysics, National Bioinformatics Infrastructure Sweden, Science for Life Laboratory, Stockholm University, Box 1031, Solna, 17121, Sweden; 6 Clinical Genetics, Department of Molecular Medicine and Surgery, Karolinska Institutet, MMK L1:00, Karolinska University Hospital at Solna, Stockholm, 171 76, Sweden; 7 Department of Medical Sciences, National Bioinformatics Infrastructure Sweden, Science for Life Laboratory, Uppsala University, Husargatan 3, Uppsala, 752 37, Sweden; 8 Department of Microbiology, Tumor and Cell Biology, Clinical Genomics Facility, Science for Life Laboratory, Karolinska Institutet, Box 1031, Solna, 171 21, Sweden; 9 School of Engineering Sciences in Chemistry, Biotechnology and Health, Science for Life Laboratory, KTH Royal Institute of Technology, Box 1031, Solna, 17121, Sweden

**Keywords:** Analysis workflow, Whole Genome Sequencing, Germline variants, Somatic variants, Cancer

## Abstract

Whole-genome sequencing (WGS) is a fundamental technology for research to advance precision medicine, but the limited availability of portable and user-friendly workflows for WGS analyses poses a major challenge for many research groups and hampers scientific progress. Here we present Sarek, an open-source workflow to detect germline variants and somatic mutations based on sequencing data from WGS, whole-exome sequencing (WES), or gene panels. Sarek features (i) easy installation, (ii) robust portability across different computer environments, (iii) comprehensive documentation, (iv) transparent and easy-to-read code, and (v) extensive quality metrics reporting. Sarek is implemented in the Nextflow workflow language and supports both Docker and Singularity containers as well as Conda environments, making it ideal for easy deployment on any POSIX-compatible computers and cloud compute environments. Sarek follows the GATK best-practice recommendations for read alignment and pre-processing, and includes a wide range of software for the identification and annotation of germline and somatic single-nucleotide variants, insertion and deletion variants, structural variants, tumour sample purity, and variations in ploidy and copy number. Sarek offers easy, efficient, and reproducible WGS analyses, and can readily be used both as a production workflow at sequencing facilities and as a powerful stand-alone tool for individual research groups. The Sarek source code, documentation and installation instructions are freely available at https://github.com/nf-core/sarek and at https://nf-co.re/sarek/.

## Introduction

Whole-genome sequencing (WGS) and whole-exome sequencing (WES) technologies opens up new avenues for research and for clinical applications, with many large initiatives launched worldwide. While much effort has been invested in novel sequencing analysis software, the importance of providing and maintaining workflows to combine software in an efficient and reproducible manner has been underestimated and too few resources are typically dedicated to address this issue. This is of particular importance for somatic variant analysis and especially for analysis of complex cancer genomes, where a combination of tools is still required for optimal sensitivity and specificity and to detect various types of gene mutations and other abnormalities ( Alioto *et al*., 2015). Some encouraging solutions have been presented in recent years, including SeqMule ( Guo *et al*., 2015), SpeedSeq ( Chiang *et al*., 2015), Bcbio-nextgen, and DNAp ( Causey *et al*., 2018). While all of the above represent commendable and important efforts, we have not found any workflow solution that in our opinion fulfils all of the following important user aspects: (i) easy installation, (ii) robust portability across different compute environments, (iii) comprehensive documentation, (iv) transparent and easy-to-read code, and (v) extensive quality metrics reporting. Here we present Sarek, an easy-to-install community-maintained workflow, offering a complete and scalable solution for germline and somatic variant detection, annotation and quality control. Sarek supports several reference genomes and can handle data from WGS, WES and gene panels, and is intended to be used both as a production workflow at core facilities and as a stand-alone tool for individual research groups. By using Docker or Singularity containers, Sarek installs easily on all POSIX compatible systems such as Linux and Mac OS X and is designed to work on compute environments dedicated to handle sensitive personal data without direct internet access—a situation expected to become increasingly common with growing data security awareness.

## Methods

### Operation: workflow overview and software

**Figure 1.**
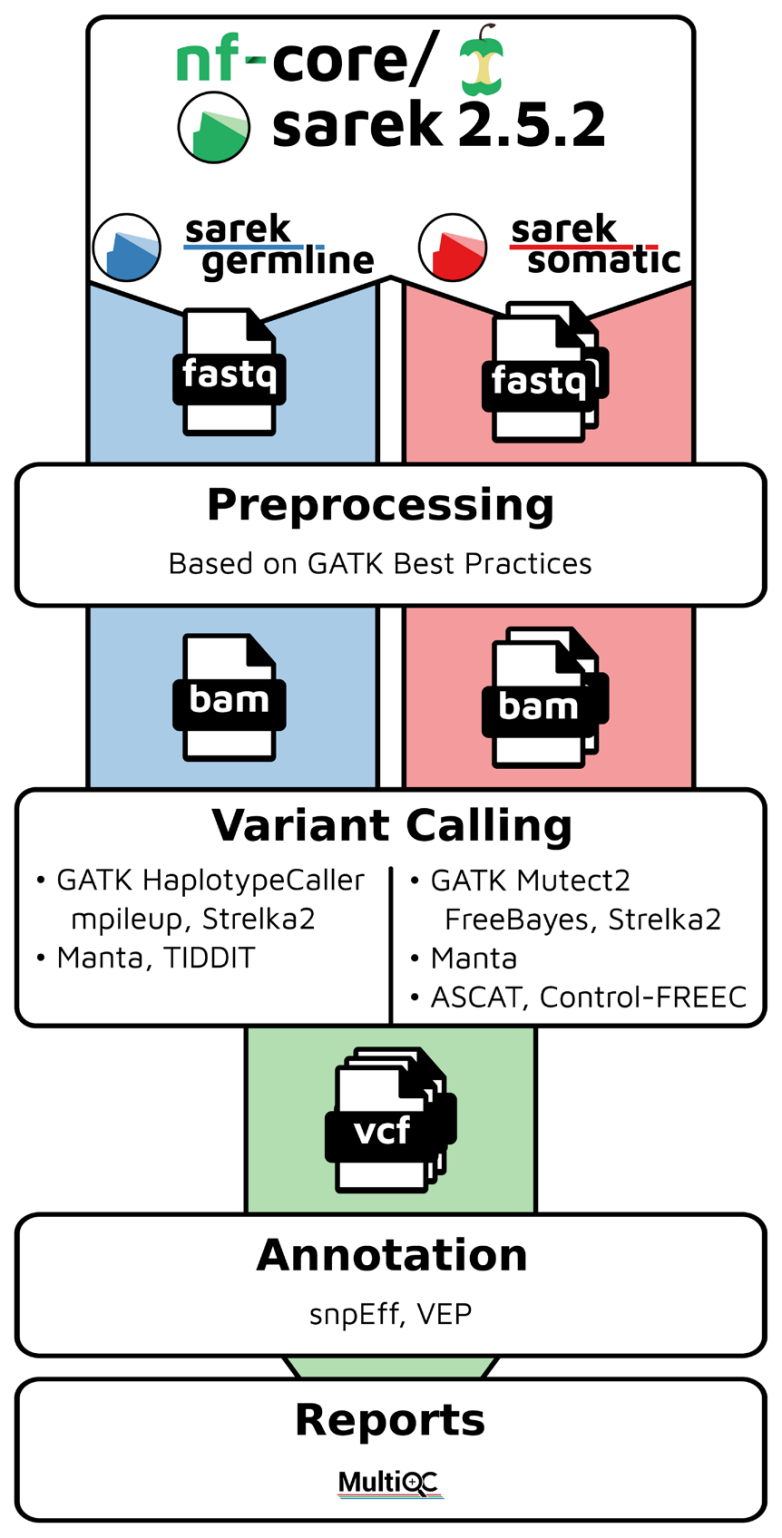
Schematic overview of the Sarek workflow for analysis of germline and somatic variants. A schematic overview including some of the main analysis software implemented in the Sarek workflow. A more comprehensive list of the currently implemented software is given in Table 1.

Sarek offers a portable workflow for germline and somatic variant detection, annotation and quality control based on WGS, WES or gene panel data, using a range of state-of-the-art software and data resources in the field ( Table 1, Figure 1). In the pre-processing step, sequence reads are aligned to the reference genome with BWA-MEM ( Li, 2013), followed by deduplication and recalibration with GATK ( McKenna *et al*., 2010). For germline samples, single-nucleotide variants and small insertion/deletions are detected with HaplotypeCaller ( McKenna *et al*., 2010) and Strelka2 ( Kim *et al*., 2018), and structural variations are detected with Manta ( Chen *et al*., 2016) and TIDDIT ( Eisfeldt *et al*., 2017). For somatic samples, somatic single-base mutations (SSM) and small somatic insertion/deletion mutations (SIM) are detected by GATK4 Mutect2 ( Cibulskis *et al*., 2013) and Strelka2 ( Kim *et al*., 2018). Somatic structural variants (including copy-number variation), as well as ploidy and sample purity are detected by Manta ( Chen *et al*., 2016), ASCAT ( Van Loo *et al*., 2010), and Control-FREEC ( Boeva *et al*., 2012). All variants are annotated for potential functional effects with snpEff ( Cingolani *et al*., 2012) and VEP ( McLaren *et al*., 2016). Importantly, Sarek also generates a wide range of quality control metrics using FastQC, QualiMap ( Okonechnikov *et al*., 2016), BCFtools ( Li, 2011), Samtools ( Li *et al*., 2009), and VCFtools ( Danecek *et al*., 2011), visualized as an aggregated quality control review across samples with MultiQC ( Ewels *et al*., 2016). All software currently included in Sarek are selected based on the criteria that they should be of high quality, well-maintained, and with robust installation and running performances. Additional alternative or complementing software will be added to Sarek in later updates, based on the input and engagement of the user community.

**Table 1.** Software required and implemented in Sarek. A list of all the software required and currently implemented in Sarek. All analysis and quality metrics software are installed automatically when Sarek is launched. P, Preprocessing; G, Germline; S, Somatic; snv, Single-nucleotide variants and small indels; sv, Structural variants; pp, Ploidy and sample purity; a, Annotation.

Software/Resource	Analyses	Availability
**Required software**		
Nextflow		https://www.nextflow.io/index.html
Docker, Singularity or Conda		https://www.docker.com/, https://sylabs.io/, https://docs.conda.io/en/latest/
**Included analysis software**		
BWA-MEM	P	http://bio-bwa.sourceforge.net/
GATK4	P, G(snv)	https://software.broadinstitute.org/gatk/
Samtools	P, G(snv)	https://github.com/samtools/samtools
Strelka2	G(snv), S(snv)	https://github.com/Illumina/strelka
Manta	G(sv), S(sv)	https://github.com/Illumina/manta
TIDDIT	G(sv)	https://github.com/SciLifeLab/TIDDIT
GATK4 Mutect2	S(snv)	https://gatk.broadinstitute.org/hc/en-us/articles/360037593851-Mutect2
Freebayes	S(snv)	https://github.com/ekg/freebayes
ASCAT	S(pp)	https://github.com/Crick-CancerGenomics/ascat
Control-FREEC	S(pp)	http://boevalab.inf.ethz.ch/FREEC/
snpEff	G(a), S(a)	http://snpeff.sourceforge.net/
VEP	G(a), S(a)	http://www.ensembl.org/vep
**Included quality metrics software**	
MultiQC		http://multiqc.info/
FastQC		https://www.bioinformatics.babraham.ac.uk/projects/fastqc/
BamQC		https://github.com/s-andrews/BamQC
QualiMap		http://qualimap.bioinfo.cipf.es/
BCFtools		https://github.com/samtools/bcftools
VCFtools		https://vcftools.github.io/index.html

### Portability and reproducibility

Sarek is implemented in Nextflow ( Di Tommaso *et al*., 2017), a workflow language designed specifically for bioinformatics applications. Nextflow has a transparent design, making the Sarek code easy to read, adjust and extend. Sarek has well-functioning error reporting to diagnose e.g. software or hardware errors during a run, and incomplete runs are easily restarted from any stage in the workflow process. Compared to the Bpipe workflow language (used in for example DNAp), Nextflow offers superior support for different execution environments, like Slurm, Sun Grid Engine, LSF and Kubernetes, and includes native support for cloud compute environments including Google Cloud and AWS. Support for AWS batch gives the possibility to easily distribute thousands of batch jobs on Amazon Web Services. Sarek is part of a rapidly growing community effort of well documented and community-tested Nextflow pipelines, and adheres to the nf-core portability and documentation guidelines ( Ewels *et al*., 2019). To facilitate easy installation and to ensure reproducibility, all Sarek required tools are installed in Conda, and then pushed to DockerHub ( https://hub.docker.com/), making Sarek and all its dependencies directly accessible from a Conda environment, or as Docker or Singularity ( Kurtzer *et al*., 2017) containers. While Docker is a widely appreciated container solution, it is not always allowed at high-performance computing centers because of the involved security risks, making Singularity the preferred choice at these sites ( Kurtzer *et al*., 2017). This is of particular importance for computer environments designed for handling of sensitive personal data, where a high level of data security has to be maintained across multiple projects and users.

### Implementation: equipment and resource usage

Sarek can be installed and executed on any POSIX-compatible computer system. To run a full WGS analysis, including both germline and somatic variants from a tumour/normal dataset with 90x/90x read coverage, we recommend a minimum of 16 cores on a node with 128 GB RAM, and at least 4 TB available free storage (in addition to the initial FASTQ files) in the input/output working directory. Of this, about 1.4 TB will be allocated for BAM files, annotated VCF files and CNV files, but excluding GVCF files ( Table 2). At the end of the run, 2.3 TB temporary data can be removed, unless the user plans to perform re-runs from intermediate processing states. Many processes are distributed across cores by dividing the genome into smaller chunks, each being handled as a separate core job, with all the results being merged and sorted in a final step. Some of the used software are parallelized by design, while for others Sarek uses a scatter-gather approach to efficiently distribute the processing load across CPU cores and reduce the wall clock runtime.

**Table 2.** Sarek resource usage. Resource usage during a Sarek run on a WGS 90X/90X coverage medulloblastoma dataset on a 48-threaded computer node, starting from compressed FASTQ files. The storage resources refer to result files only. The total storage including all temporary data was 3.7 TB.

	Input data	Mapping, merging, deduplication	Quality score recalibration	Variant calling, annotation	Total
**Storage**	458 GB	530 GB	386 GB	4 GB	1378 GB
**Process time**		1081 CPU h	95 CPU h	614 CPU h	1790 CPU h
**Wall clock time**		35h 26m	3h 26m	13h 29m	48h 21m
**Peak memory**		119 GB	18 GB	128 GB	

GB, gigabyte; CPU, central processing unit; h, hours; m, minutes.

### Installation and testing

Sarek is run from a computer system with a local installation of Nextflow and support for either Conda environments, Docker or Singularity containers. Nextflow can automatically fetch the Sarek source code from GitHub. All software dependencies are encapsulated in Docker or Singularity containers which are downloaded from Docker Hub, or built in a new Conda environment using Bioconda ( Grüning *et al*., 2018). As such, cumbersome software installations by the user are completely avoided. Configuration files allow tailoring to specific user needs. Sarek comes with a small test dataset and a suite of tests to verify the installation. This is also used for Continuous Integration testing with GitHub Actions.

## Results

To test performance in terms of resource usage and biological results, Sarek was run on a medulloblastoma WGS tumour/normal dataset from a sample with high tumour cell content (∼98%), and with a curated “Gold Set” of verified somatic mutations from a previous benchmark study ( Alioto *et al*., 2015). In line with the above benchmark study, Sarek (version 2.5.2) was executed with WGS germline and somatic variant calling using a 90X/90X tumour/normal dataset (accession number EGAD00001001859, read sets EGAR00001387019-24 and EGAR00001387025-32). Runs were performed on a single 48-thread node with a local direct attached storage (DAS): A Dell PowerEdge R740 server, with two Intel Xeon Gold 6126 with a total of 24 cores (48 threads) CPUs, 756 GB memory, and 100 TB SCv3020 Compellent Storage. The complete Sarek run including preprocessing followed by both germline and somatic variant calling and annotation took 48 hours and 21 minutes, and required about three times more storage than the original input data ( Table 2). Notably, the complete Sarek run was executed by a single command, with fully automated installation, execution, and efficient job distributions of the more than 15 different software tools to complete the analysis and provide quality control metrics, without any manual intervention needed during the two-day run. To ensure that the Sarek output was biologically sound, we calculated precision, recall and F1 statistics for the Sarek output based on the “Gold Set” of somatic single-base mutations (SSM) and somatic insertion/deletion mutations (SIM) as previously defined ( Alioto *et al*., 2015). Using the intersection of the output from the two somatic variant callers (GATK4 Mutect2 and Strelka2), Sarek provided accuracy measures for SSMs (F1 score = 0.80) and SIMs (F1 score = 0.58) in the top range of the 18 somatic variant calling procedures included in the original benchmarking study on this data set ( Table 3), indicating that the workflow operates as intended. The sample purity was estimated to be 100%, as compared to 98% previously reported for this sample. For somatic structural variants and ploidy, no relevant benchmark data was available, and therefore no quantitative assessment beyond previously published results for the implemented software could be performed, but the integrity of the runs were checked by comparing the results of Manta, ASCAT, and Control-FREEC run within Sarek and as stand-alone. To benchmark Sarek on germline single-nucleotide variants and small insertions/deletions, we used 46X WGS data for the well-studied individual NA12878:HG001 ( ftp://ftp-trace.ncbi.nlm.nih.gov/giab/ftp/data/NA12878/NIST_NA12878_HG001_HiSeq_300x/, read set folders 131219_D00360_005_BH814YADXX [accession number SRR2052337 - SRR2052339, SRR2052342, SRR2052345, SRR20523428], and 131219_D00360_006_AH81VLADXX [accession number SRX1049774 -SRX1049779]) and a “Gold Set” of variants from the Genome in a Bottle project ( Zook *et al.*, 2019), showing overall high accuracy ( Table 4).

**Table 3.** Sarek WGS somatic variant benchmarking. Summary of accuracy measures for the two somatic variant callers used in Sarek to detect somatic single-base mutations (SSMs) and somatic insertion/deletion mutations (SIMs), as well as their union and intersection.

Somatic caller	Recall	Precision	F1-score
**SSM (Gold Set: n=1263)**			
GATK4 Mutect2	0.80	0.45	0.58
Strelka2	0.77	0.29	0.42
Union (GATK4 Mutect2, Strelka2)	0.82	0.23	0.36
Intersection (GATK4 Mutect2, Strelka2)	0.74	0.88	0.80
Benchmark median *	0.68	0.78	0.71
**SIM (Gold Set: n=347)**			
GATK4 Mutect2	0.48	0.38	0.42
Strelka2	0.74	0.31	0.44
Union (GATK4 Mutect2, Strelka2)	0.77	0.25	0.38
Intersection (GATK4 Mutect2, Strelka2)	0.46	0.77	0.58
Benchmark median *	0.34	0.71	0.48

* The median accuracy measures across 18 somatic variant calling procedures as previously reported ( Alioto *et al*., 2015)

**Table 4.** Sarek WGS germline variant benchmarking. Summary of accuracy measures for the two variant callers used in Sarek to detect germline single-nucleotide variants (SNVs) and germline insertion/deletion variants (INDELs), as well as their union and intersection.

Germline caller	Recall	Precision	F1-score
**SNV (Gold Set: n=3088156)**			
GATK4 HaplotypeCaller	0.93	1.00	0.96
Strelka2	0.98	1.00	0.99
Union (GATK4 HaplotypeCaller, Strelka2)	0.99	0.94	0.96
Intersection (GATK4 HaplotypeCaller, Strelka2)	0.93	1.00	0.96
**INDEL (Gold Set: n=530423)**			
GATK4 HaplotypeCaller	0.91	0.99	0.95
Strelka2	0.92	0.99	0.95
Union (GATK4 HaplotypeCaller, Strelka2)	0.93	0.98	0.96
Intersection (GATK4 HaplotypeCaller, Strelka2)	0.90	1.00	0.94

## Use case

Sarek has been extensively tested and applied on various WGS datasets, including thousands of samples for germline variant analyses, and hundreds of paired tumour/normal samples for somatic mutation analyses. In addition, Sarek has also been adapted to run on WES data and gene panels, and has been reported to work well in pilot user projects, although no systematic testing has yet been performed on such data. Below we present a standard use case with a tumour/normal WGS dataset as input, running both germline and somatic variant analyses.

### Input data

For a somatic variant analysis, the user should provide the sequencing FASTQ files from both tumour and normal control tissue from the same individual, described in a tab-delimited TSV file (here: *samples.tsv*). Each line of the TSV file contains information about a sequence data file, including: The identifier of the individual, the gender (XX or XY), the status of the sample (0 for Normal or 1 for Tumour), the identifier of the sample, the sequencing lane (if samples are multiplexed across multiple lanes), and the paths to the FASTQ file of the first and second read in the read-pair. Relapse samples from the same individual are also supported.

### Running sarek on WGS data with singularity containers

Running Sarek with Singularity container on a computer system supporting Java 8 requires only installation of Nextflow and Singularity. A full analysis run starting from FASTQ files including mapping, recalibration, variant calling and annotation, as well as generating a full QC report can be invoked by a single Nextflow command:


> nextflow run nf-core/sarek -r 2.5.2 -profile singularity --input samples.tsv --tools Mutect2,Strelka,Manta,TIDDIT,ASCAT,ControlFREEC,snpEff,VEP


Nextflow will recognize the workflow name and will download the specified version (2.5.2) of the pipeline from GitHub, including the corresponding container, as well as fetching the required reference files from AWS-iGenomes. The default reference genome is human GRCh38, but Sarek also supports GRCh37 and nearly 30 other genomes directly accessible from iGenomes. Alternatively, users can manually supply Sarek with other reference genomes. Non-default parameters and links to local reference files are handled in accordance with nf-core guidelines. User configuration profiles can be stored locally or centrally at https://github.com/nf-core/configs.

### Output

A full Sarek run will produce a large number of output files, but the main results consist of (i) a set of annotated variants in VCF files from the various included tools for both germline and somatic variants, (ii) tumour sample purity and ploidy results for somatic samples, and (iii) a broad set of QC metrics. A detailed description of all output files is given at the Sarek documentation pages. While Sarek will report variants from all callers included in the run, it is up to the user to decide how to combine and filter the results from different callers, since the optimal post-processing will depend on the particular samples and research questions at hand.

## Discussion

Human WGS is transforming medical research, and provides a foundation to develop novel clinical applications and improve health care. An important aspect to harvesting the potential of WGS is however to empower the research community with adequate bioinformatics tools, and reproducible bioinformatics workflows are important drivers of scientific progress by making complex processing of large datasets feasible for a wide range of researchers. While we are highly appreciative of existing workflows for cancer and non-cancer variant detection, we argue that there is no one-size-fits-all solution and more initiatives are needed to serve the large and diverse research user community, especially for WGS data. Sarek builds on a philosophy of reasonably narrow, independent workflows, written in the domain-specific language Nextflow. In our experience, this is an effective strategy to simplify workflow maintenance at sequencing core facilities, and to allow easy deployment and modifications by individual research groups. Sarek efficiently utilizes cloud and high-performance compute clusters and installs easily across compute environments. Sarek provides annotated VCF files, CNV reports and quality metrics for germline and cancer samples from raw FASTQ sequencing data in about 48 hours for 90X/90X WGS data (as demonstrated here), in a few hours for WES data, and within minutes for gene panels (in-house data, not presented here). It should be noted that while Sarek can substantially reduce the labor and management time of running and maintaining a large collection of software, and help users to perform quality-controlled reporting in an organized manner, careful parameter tuning, downstream variant filtering, and qualitative assessments by the user remains important. Ongoing efforts aim to develop add-on ranking and visualization modules and to efficiently extract clinically and biologically relevant findings, to help advance basic and translational research.

## Conclusion

Sarek is a portable and reproducible workflow to detect germline and somatic variants from WGS, WES and gene panel data. It includes extensive analysis and quality control metrics, while still being limited to a relatively narrow scope to achieve optimal usability, functionality and transparency. Sarek is flexible with a low threshold for user modifications, and is thus well adapted to the current requirements in the research community. Thanks to its design, it installs easily and reproducibly on all POSIX compatible computer systems, including secure compute environments for sensitive personal data with indirect Internet access.

## Data availability

### Source data

European Genome-phenome Archive: A comprehensive assessment of somatic mutation detection in cancer using whole genome sequencing. https://www.ebi.ac.uk/ega/datasets/EGAD00001001859. Read sets EGAR00001387019-24 and EGAR00001387025-32 were analysed. These data are held under restricted access. Readers wishing to apply for access to the data must first apply through the ICGC Data Access Compliance Office ( https://icgc.org/daco) and complete the data access form. Access will be granted to those whose projects conform to the goals and policies of ICGC. Help with completing the data access form is available at https://icgc.org/daco/help-guide-section.

Sequence Read Archive: NIST Genome in a Bottle, ^~^300X sequencing of HG001 (NA12878). ftp://ftp-trace.ncbi.nlm.nih.gov/giab/ftp/data/NA12878/NIST_NA12878_HG001_HiSeq_300x/, read set folders 131219_D00360_005_BH814YADXX [SRA accession number SRR2052337 - SRR2052339, SRR2052342, SRR2052345, SRR20523428], and 131219_D00360_006_AH81VLADXX [SRA accession number SRX1049774 -SRX1049779]). These data are publicly available for direct download.

The workflow itself comes with a prebuilt profile with a complete configuration for automated testing, including links to a small test dataset.

## Software availability

**Sarek is available at:** https://nf-co.re/sarek.

**Source code available at:** https://github.com/nf-core/sarek.

**Archived source code at time of publication:** https://doi.org/10.5281/zenodo.3579102 ( Garcia *et al*., 2019).

**License:** MIT License.
