# Supplementary material for: Sarek: A portable workflow for whole-genome sequencing analysis of germline and somatic variants
Source: F1000Res. 2020 Sep 4;9:63. Originally published 2020 Jan 29. [Version 2] doi: 10.12688/f1000research.16665.2 (PMC7111497; doi:10.12688/f1000research.16665.2)
Supplement: Peer review history

# Peer review: Reviewer response for version 1

**DOI:** 10.5256/f1000research.18214.r61129

**Published:** 2020-03-31

**Authors:** Esa Pitkänen

**Affiliations:** 1 Institute for Molecular Medicine Finland (FIMM), Helsinki Institute of Life Science, University of Helsinki, Helsinki, Finland; 2 Applied Tumor Genomics Research Program, Faculty of Medicine, University of Helsinki, Helsinki, Finland

This manuscript describes Sarek, a workflow for analyzing next-generation sequencing (NGS) data. Sarek is based on Nextflow, a popular tool for defining computational workflows. In order to process NGS data, i.e., generating annotated variant calls ready for downstream analyses, multiple complex software tools need to be executed. This is not only computationally demanding, but also labor-intensive due to operators having to install and maintain a complicated collection of software as well as diagnose failed analysis runs, often resulting in high management overhead compared to the total computation time (Yakneen and Waszak, 2020 ^1^). Sarek aims to minimize the installation and management time overhead by building a NGS workflow on top of Nextflow, automatically installing the required software components. These software consist of some of the state-of-the-art tools in read mapping, variant calling and annotation and quality control. Sarek is a welcome addition to the toolkit of bioinformaticians looking for an NGS analysis workflow, which can be easily installed on a HPC cluster or cloud environment. The article is well-written and clear to understand. While I'm happy to recommend indexing of the manuscript also in the present form, I have a few suggestions how to improve it:

**Major comments:**

1. While some of the existing NGS workflows are mentioned, I would appreciate it if Sarek was compared to these approaches in more detail. Is there functionality that is currently missing from Sarek that is present in one of the other workflows?
2. Typically in NGS data analysis, a lot of time can be spent on debugging failed runs to find out whether one of the tools failed, if the data is corrupted/missing or if there was a hardware error. How does Sarek support run diagnostics and relaunching failed jobs?
3. How does Sarek combine variant calls when multiple callers are used for a variant type? 
4. Somatic single nucleotide and indel variant calls from Sarek were shown to match well with a previously defined gold standard callset. No benchmark data was available for more complex somatic variants and variant calling accuracy for germline variants was not evaluated. I am interested in seeing more comprehensive tests to cover all germline and somatic variant types.
5. I would appreciate it if a minimal test dataset together with instructions and a suite of automated tests was provided with Sarek. This would make it easier for the user to test out an installation as well as raising issues in GitHub. 

 

**Minor comments:**

1. How easy it is for users to modify or extend Sarek by for example adding a new variant caller to the workflow? This could be explored in more detail in text.
2. It would be good to easily see which tools are used to call and analyze each variant type. This information could be added either to Fig 1, Table 1 or both.
3. FreeBayes is included in Figure 1 but missing from Table 1.
4. The wording in “To facilitate easy installation and to ensure reproducibility, all Sarek required tools are managed in Docker or Singularity (Kurtzer et al., 2017) containers, or a Conda environment.” should be clarified -- are all tools being maintained in all the three systems?
5. When running Sarek with default options, it crashes in the tool version check. It took me a while to figure out this was due to the default “-profile” argument of “standard” which seems to assume Singularity is available. It would be good to improve error messages so that it is easier to understand the underlying cause. A minimal installation and testing procedure mentioned above would help in this regard.
6. Typo: “Whole-genome sequencing (WGS) and whole-exome sequencing (WES) technologies opens…” -> “...open”.

Are the conclusions about the tool and its performance adequately supported by the findings presented in the article?

Yes

Is the rationale for developing the new software tool clearly explained?

Yes

Is the description of the software tool technically sound?

Yes

Are sufficient details of the code, methods and analysis (if applicable) provided to allow replication of the software development and its use by others?

Yes

Is sufficient information provided to allow interpretation of the expected output datasets and any results generated using the tool?

Yes

Reviewer Expertise:

Bioinformatics, cancer genetics, machine learning

I confirm that I have read this submission and believe that I have an appropriate level of expertise to confirm that it is of an acceptable scientific standard.

## References

[1] : Butler enables rapid cloud-based analysis of thousands of human genomes. *Nat Biotechnol*.2020;38(3) : 10.1038/s41587-019-0360-3 288-292 10.1038/s41587-019-0360-3 32024987PMC7062635

# Response

**Published:** 2020-07-01

**Authors:** Björn Nystedt

**Affiliations:** Uppsala university, Sweden

**We are grateful to the reviewer for the positive and constructive comments! We have uploaded a revised version of the manuscript and included updated documentation for the workflow, including adjustments and improvements as suggested by the reviewer, and as described in the detailed comments below, marked in bold.**

 Approved

 This manuscript describes Sarek, a workflow for analyzing next-generation sequencing (NGS) data. Sarek is based on Nextflow, a popular tool for defining computational workflows. In order to process NGS data, i.e., generating annotated variant calls ready for downstream analyses, multiple complex software tools need to be executed. This is not only computationally demanding, but also labor-intensive due to operators having to install and maintain a complicated collection of software as well as diagnose failed analysis runs, often resulting in high management overhead compared to the total computation time (Yakneen and Waszak, 2020 ^1^). Sarek aims to minimize the installation and management time overhead by building a NGS workflow on top of Nextflow, automatically installing the required software components. These software consist of some of the state-of-the-art tools in read mapping, variant calling and annotation and quality control. Sarek is a welcome addition to the toolkit of bioinformaticians looking for an NGS analysis workflow, which can be easily installed on a HPC cluster or cloud environment. The article is well-written and clear to understand. While I'm happy to recommend indexing of the manuscript also in the present form, I have a few suggestions how to improve it:

 Major comments:

1. While some of the existing NGS workflows are mentioned, I would appreciate it if Sarek was compared to these approaches in more detail. Is there functionality that is currently missing from Sarek that is present in one of the other workflows?

**This is a relevant comment, but a detailed comparison of the current functionality of different workflows risk being quickly outdated as functionality will frequently change both in Sarek and in the other mentioned workflows. Also, the main purpose of Sarek is not to provide unique functionality *per se*, but to provide a workflow solution with some generally important features as detailed in the manuscript**

***“..(i) easy installation, (ii) robust portability across different compute environments, (iii) comprehensive documentation, (iv) transparent and easy-to-read code, and (v) extensive quality metrics reporting.”***

**Therefore, the main difference between Sarek and the other mentioned workflows is in practical usability rather than a particular functionality, as these can typically be tuned or added as needed. **

1. Typically in NGS data analysis, a lot of time can be spent on debugging failed runs to find out whether one of the tools failed, if the data is corrupted/missing or if there was a hardware error. How does Sarek support run diagnostics and relaunching failed jobs?

**This is a good comment, and NextFlow reports the failed process and the error-code from the underlying software, and Sarek is designed to make it very easy to resume failed jobs from the point of failure. This has now been better highlighted in the manuscript under the heading “Portability and reproducibility”. We will work continuously with the user community to further avoid run failures and to continuously improve the diagnostic capability and error-handling.**

1. How does Sarek combine variant calls when multiple callers are used for a variant type? 

**Sarek will report variants from all callers include in the run, but it is up to the user to decide on how to combine results from different callers, since e.g. the optimal balance between high specificity *versus* high sensitivity differs across research projects. This has been clarified in the manuscript under the heading “Output”, and in the “Discussion”.   **

1. Somatic single nucleotide and indel variant calls from Sarek were shown to match well with a previously defined gold standard callset. No benchmark data was available for more complex somatic variants and variant calling accuracy for germline variants was not evaluated. I am interested in seeing more comprehensive tests to cover all germline and somatic variant types.

**This is a good suggestion and we have run germline variant calling with Sarek on the HG001 sample and compared to the Genome In a Bottle gold standard dataset, and these results are now included in the manuscript.**

**Benchmarking of complex somatic variants is very complex and difficult due to the lack of robust and relevant benchmark datasets making such testing beyond the scope of this publications, since Sarek is a workflow and does not provide any novel algorithms or methodology *per se*. For now, we refer users to the tests published along with the respective included variant calling software. This limitation is stated in the manuscript under the heading “Results”.**


**  **

1. I would appreciate it if a minimal test dataset together with instructions and a suite of automated tests was provided with Sarek. This would make it easier for the user to test out an installation as well as raising issues in GitHub. 

**This is a good suggestion, and there is actually already a minimal test dataset and a suit of tests available and documented in Sarek, as detailed under the heading “Installation and testing”. We have improved the Sarek documentation to make this clear and easy to find.**

 Minor comments:

1. How easy it is for users to modify or extend Sarek by for example adding a new variant caller to the workflow? This could be explored in more detail in text.

**This is a good suggestion and we have clarified this point in the manuscript under the heading “Operation: Workflow overview and software”. In brief, we have started with software we have judged being of high quality, well-maintained and robust. Additional software will be added to Sarek later on in a community effort, and this process is already ongoing.**


**  **

1. It would be good to easily see which tools are used to call and analyze each variant type. This information could be added either to Fig 1, Table 1 or both.

**This is a good suggestion and we have added this information to Table 1.**


**  **

1. FreeBayes is included in Figure 1 but missing from Table 1.

**This is a good note, and we have adjusted the manuscript accordingly. FreeBayes can optionally be run in Sarek, and it is now included in Table 1.**


**  **

1. The wording in “To facilitate easy installation and to ensure reproducibility, all Sarek required tools are managed in Docker or Singularity (Kurtzer et al., 2017) containers, or a Conda environment.” should be clarified -- are all tools being maintained in all the three systems?

**This is a very useful comment, and this has now been clarified in the manuscript under the heading “Portability and reproducibility”. All tools are installed in Conda, and then pushed to DockerHub (https://hub.docker.com/). This way all tools are available directly from all three systems; Conda, Docker and Singularity.**


**  **

1. When running Sarek with default options, it crashes in the tool version check. It took me a while to figure out this was due to the default “-profile” argument of “standard” which seems to assume Singularity is available. It would be good to improve error messages so that it is easier to understand the underlying cause. A minimal installation and testing procedure mentioned above would help in this regard.

**This is a very useful comment, and we have improved the error message and the documentation regarding the “-profile” arguments. **

1. Typo: “Whole-genome sequencing (WGS) and whole-exome sequencing (WES) technologies opens…” -> “...open”.

**This typo has been corrected in the manuscript. **

- Is the rationale for developing the new software tool clearly explained?

Yes

- Is the description of the software tool technically sound?

Yes

- Are sufficient details of the code, methods and analysis (if applicable) provided to allow replication of the software development and its use by others?

Yes

- Is sufficient information provided to allow interpretation of the expected output datasets and any results generated using the tool?

Yes

- Are the conclusions about the tool and its performance adequately supported by the findings presented in the article?

Yes

# Peer review: Reviewer response for version 1

**DOI:** 10.5256/f1000research.18214.r59295

**Published:** 2020-03-09

**Authors:** Tony Håndstad

**Affiliations:** 1 Oslo University Hospital, Oslo, Norway

Sarek is a workflow for variant detection and analysis of sequencing data from WGS, WES and targeted panels. The workflow is comprehensive and versatile, allowing for variant detection in both germline and somatic samples, from WGS/WES/panel sequencing.

- It includes variant calling of SNPs, indels, and structural variants, as well as annotation and extensive quality control.
- Sarek is open source and part of the nf-core community effort which builds well-curated analysis pipelines in the Nextflow pipeline framework.
- Sarek is very user friendly, and installation, configuration and execution is easy to perform, while the workflow is also flexible.
- Implementation manages to be clear, despite also being advanced with parallelization and ample choice of installation/execution. Many researchers would likely struggle to implement pipelines at this advanced level.
- The documentation is excellent, and despite the comprehensive functionality, most users should find it easy to set up Sarek and get started. I have no doubt that Sarek will be a very valuable addition to the research community.

     

 The paper is well written and fulfils all the reviewer criteria. As reviewer, I have only a few minor comments:

- Sarek can use different Nextflow configuration profiles. In the Sarek documentation, it says that the test profile is a profile with a complete configuration for automated testing and that it includes links to test data so needs no other parameters.
  It should be obvious to most users, but I would suggest that the authors make it clear that when using the test profile, a user must also supply conda, docker or singularity profile if not all the tools are installed in the PATH. This is clear from the general nf-core documentation (https://nf-co.re/usage/introduction) but less so from the Sarek documentation.

- Whereas the choice of Nextflow is justified, there is little argumentation for why the different tools (variant callers in particular) are selected other than that they represent state-of-the-art. Also why several tools for variant calling are combined is not mentioned, though the referred paper (Alioto *et al.*, 2015) makes a clear case for this, at least for somatic variant calling.

- The paper title makes it clear that Sarek is for whole genome sequencing analysis, but as stated in the text, Sarek is also applicable to exome and targeted panel analyses where the authors say it has been run successfully.
  Many researchers are using exome sequencing, so it could be of interest to know if the authors have an opinion or experience with how use of targeted sequencing data limit Sarek in terms of accuracy or utility, for example, are the tools used for structural variant calling able to handle exome data well (to the extent possible with targeted sequencing?)
  The documentation has a small chapter stating that the authors recommend supplying a BED file with the targeted regions, but there is not so much explanation of what the effect of this is.

- The authors demonstrate that Sarek is both fast and accurate by running it on a tumor/normal(germline) dataset from a previous benchmark study. I think this is acceptable/sufficient, but one could always wish for more; the paper could be strengthened by for example running the well-known public germline HG001 sample against the Genome In a Bottle gold standard dataset.
- Accurate somatic variant calling is difficult. But the included benchmark study demonstrates that Sarek performs well in comparison with other pipelines. The tool leaves it up to the user to decide whether to use output from a single variant caller or the union or intersection from all tools for increased sensitivity or precision.
- In summary, I think Sarek is a great addition to the community and recommend the paper for indexing.

Are the conclusions about the tool and its performance adequately supported by the findings presented in the article?

Yes

Is the rationale for developing the new software tool clearly explained?

Yes

Is the description of the software tool technically sound?

Yes

Are sufficient details of the code, methods and analysis (if applicable) provided to allow replication of the software development and its use by others?

Yes

Is sufficient information provided to allow interpretation of the expected output datasets and any results generated using the tool?

Yes

Reviewer Expertise:

Diagnostic bioinformatics (variant calling pipelines) and variant interpretation

I confirm that I have read this submission and believe that I have an appropriate level of expertise to confirm that it is of an acceptable scientific standard.

# Response

**Published:** 2020-07-01

**Authors:** Björn Nystedt

**Affiliations:** Uppsala university, Sweden

**We are grateful to the reviewer for the positive and constructive comments! We have uploaded a revised version of the manuscript and included updated documentation for the workflow, including adjustments and improvements as suggested by the reviewer, and as described in the detailed comments below, marked in bold.**

 Approved

 Sarek is a workflow for variant detection and analysis of sequencing data from WGS, WES and targeted panels. The workflow is comprehensive and versatile, allowing for variant detection in both germline and somatic samples, from WGS/WES/panel sequencing.

- It includes variant calling of SNPs, indels, and structural variants, as well as annotation and extensive quality control.
- Sarek is open source and part of the nf-core community effort which builds well-curated analysis pipelines in the Nextflow pipeline framework.
- Sarek is very user friendly, and installation, configuration and execution is easy to perform, while the workflow is also flexible.
- Implementation manages to be clear, despite also being advanced with parallelization and ample choice of installation/execution. Many researchers would likely struggle to implement pipelines at this advanced level.
- The documentation is excellent, and despite the comprehensive functionality, most users should find it easy to set up Sarek and get started. I have no doubt that Sarek will be a very valuable addition to the research community.

     

 The paper is well written and fulfils all the reviewer criteria. As reviewer, I have only a few minor comments:

- Sarek can use different Nextflow configuration profiles. In the Sarek documentation, it says that the test profile is a profile with a complete configuration for automated testing and that it includes links to test data so needs no other parameters.
  It should be obvious to most users, but I would suggest that the authors make it clear that when using the test profile, a user must also supply conda, docker or singularity profile if not all the tools are installed in the PATH. This is clear from the general nf-core documentation (https://nf-co.re/usage/introduction) but less so from the Sarek documentation.

**This is a good suggestion and we have highlighted and improved this information in the Sarek documentation. We are also working to revise the general documentation format in nf-core to make this more transparent throughout. **

- Whereas the choice of Nextflow is justified, there is little argumentation for why the different tools (variant callers in particular) are selected other than that they represent state-of-the-art. Also why several tools for variant calling are combined is not mentioned, though the referred paper (Alioto *et al.*, 2015) makes a clear case for this, at least for somatic variant calling.

**This is a good suggestion and we have clarified this point in the manuscript. In brief, we have included software we have judged being of high quality, well-maintained and robust. Additional software will be added to Sarek later on in a community effort, and this process is already ongoing. **

- The paper title makes it clear that Sarek is for whole genome sequencing analysis, but as stated in the text, Sarek is also applicable to exome and targeted panel analyses where the authors say it has been run successfully.
  Many researchers are using exome sequencing, so it could be of interest to know if the authors have an opinion or experience with how use of targeted sequencing data limit Sarek in terms of accuracy or utility, for example, are the tools used for structural variant calling able to handle exome data well (to the extent possible with targeted sequencing?)
  The documentation has a small chapter stating that the authors recommend supplying a BED file with the targeted regions, but there is not so much explanation of what the effect of this is.

**This is a relevant comment, and we want to clarify that Sarek has been run on whole-exome sequencing data in pilot user projects, and has been reported to us to work well (personal communication), but no comprehensive benchmark has been performed by us to evaluate this. This has been clarified in the Sarek documentation and in the manuscript. **

- The authors demonstrate that Sarek is both fast and accurate by running it on a tumor/normal(germline) dataset from a previous benchmark study. I think this is acceptable/sufficient, but one could always wish for more; the paper could be strengthened by for example running the well-known public germline HG001 sample against the Genome In a Bottle gold standard dataset.

**This is a good suggestion and we have run germline variant calling with Sarek on the HG001 sample and compared to the Genome In a Bottle gold standard dataset, and these results are now included in the manuscript.**

  

- Accurate somatic variant calling is difficult. But the included benchmark study demonstrates that Sarek performs well in comparison with other pipelines. The tool leaves it up to the user to decide whether to use output from a single variant caller or the union or intersection from all tools for increased sensitivity or precision.
- In summary, I think Sarek is a great addition to the community and recommend the paper for indexing.

- Is the rationale for developing the new software tool clearly explained?

Yes

- Is the description of the software tool technically sound?

Yes

- Are sufficient details of the code, methods and analysis (if applicable) provided to allow replication of the software development and its use by others?

Yes

- Is sufficient information provided to allow interpretation of the expected output datasets and any results generated using the tool?

Yes

- Are the conclusions about the tool and its performance adequately supported by the findings presented in the article?

Yes
